# Community-based asthma assessment in young children: adaptations for a multicentre longitudinal study in South Asia

**DOI:** 10.1177/20499361221103876

**Published:** 2022-07-18

**Authors:** Mohammad Shahidul Islam, Samin Huq, Steven Cunningham, Jurgen Schwarze, A.S.M.D. Ashraful Islam, Mashal Amin, Farrukh Raza, Radanath Satpathy, Pradipta Ranjan Rauta, Salahuddin Ahmed, Hana Mahmood, Genevie Fernandes, Benazir Baloch, Imran Nisar, Sajid Soofi, Pinaki Panigrahi, Sanjay Juvekar, Ashish Bavkedar, Abdullah H. Baqui, Senjuti Saha, Harry Campbell, Aziz Sheikh, Harish Nair, Samir K. Saha, RESPIRE

**Affiliations:** Usher Institute, College of Medicine & Veterinary Medicine, The University of Edinburgh, Edinburgh, UK; Child Health Research Foundation, Dhaka, Bangladesh; Child Health Research Foundation, Dhaka, Bangladesh; Child Life and Health, Centre for Inflammation Research, The University of Edinburgh, Edinburgh, UK; Child Life and Health, Centre for Inflammation Research, The University of Edinburgh, Edinburgh, UK; Projahnmo Research Foundation, Dhaka, Bangladesh; Aga Khan University, Karachi, Pakistan; Aga Khan University, Karachi, Pakistan; AIPH University, Bhubaneswar, India; AIPH University, Bhubaneswar, India; Usher Institute, College of Medicine & Veterinary Medicine, The University of Edinburgh, Edinburgh, UK; Projahnmo Research Foundation, Dhaka, Bangladesh; Neoventive Solutions, Islamabad, Pakistan; NIHR Global Health Research Unit on Respiratory Health (RESPIRE), Edinburgh, UK; Aga Khan University, Karachi, Pakistan; Aga Khan University, Karachi, Pakistan; Aga Khan University, Karachi, Pakistan; Georgetown University Medical Center, Washington, DC, USA; KEM Hospital Research Centre, Pune, India; KEM Hospital Research Centre, Pune, India; Johns Hopkins Bloomberg School of Public Health, Baltimore, MD, USA; Child Health Research Foundation, Dhaka, Bangladesh; Usher Institute, College of Medicine & Veterinary Medicine, The University of Edinburgh, Edinburgh, UK; Usher Institute, College of Medicine & Veterinary Medicine, The University of Edinburgh, Edinburgh, UK; Usher Institute, College of Medicine & Veterinary Medicine, The University of Edinburgh, Old Medical School, Teviot Place, Edinburgh, EH8 9AG, UK; Child Health Research Foundation, 23/2 Khilji Road, Dhaka 1207, Bangladesh; NIHR Global Health Research Unit on Respiratory Health (RESPIRE), Edinburgh, UK

**Keywords:** asthma, children, poor resource settings, RSV, South Asia

## Abstract

**Background:**

Systematic assessment of childhood asthma is challenging in low- and middle-income country (LMIC) settings due to the lack of standardised and validated methodologies. We describe the contextual challenges and adaptation strategies in the implementation of a community-based asthma assessment in four resource-constrained settings in Bangladesh, India, and Pakistan.

**Method:**

We followed a group of children of age 6–8 years for 12 months to record their respiratory health outcomes. The study participants were enrolled at four study sites of the ‘Aetiology of Neonatal Infection in South Asia (ANISA)’ study. We standardised the research methods for the sites, trained field staff for uniform data collection and provided a ‘Child Card’ to the caregiver to record the illness history of the participants. We visited the children on three different occasions to collect data on respiratory-related illnesses. The lung function of the children was assessed in the outreach clinics using portable spirometers before and after 6-minute exercise, and capillary blood was examined under light microscopes to determine eosinophil levels.

**Results:**

We enrolled 1512 children, 95.5% (1476/1512) of them completed the follow-up, and 81.5% (1232/1512) participants attended the lung function assessment tests. Pre- and post-exercise spirometry was performed successfully in 88.6% (1091/1232) and 85.7% (1056/1232) of children who attempted these tests. Limited access to health care services, shortage of skilled human resources, and cultural diversity were the main challenges in adopting uniform procedures across all sites. Designing the study implementation plan based on the local contexts and providing extensive training of the healthcare workers helped us to overcome these challenges.

**Conclusion:**

This study can be seen as a large-scale feasibility assessment of applying spirometry and exercise challenge tests in community settings of LMICs and provides confidence to build capacity to evaluate children’s respiratory outcomes in future translational research studies.

## Background

Asthma is a common chronic respiratory disorder that affects an estimated 334 million people worldwide,^1^ and approximately 14% of global children have asthma.^2,3^ Children with asthma have intermittently narrowed bronchial airways that obstruct airflow in the lung, making breathing difficult and triggering symptoms like coughing, wheezing, and shortness of breath.^4^ Diagnosis of asthma in children and adults is difficult due to the lack of a specific gold-standard diagnostic test.^5^ Recent reports suggest that 20–70% of asthma cases remain undiagnosed and untreated, while 30–35% of asthma cases are wrongly diagnosed and overtreated.^6–11^ The European Respiratory Society recommends doing bronchodilator reversibility (BDR) test for children aged 5–16 years under investigation for asthma to reduce the rate of asthma misdiagnosis.^11^ In the past, low- and middle-income countries (LMICs) faced obstacles in implementing asthma diagnostic guidelines. Consequently, setting operational definitions for childhood asthma was common in research conducted in LMICs and a very few studies included objective measurements in their asthma assessment protocol.^12–14^

We conducted a community-based asthma assessment in a population-based prospective cohort study in children aged 6–8 years. This study followed up a previously enrolled birth cohort (ANISA study) to assess the long-term effect of respiratory syncytial virus (RSV) infection in early infancy.^15^ Here, we described our research approach, surveillance outcomes, challenges, and adaptations necessary for the community-based asthma assessment to improve asthma diagnosis in children in poor resource settings of LMICs.

### Research context

RSV is estimated to cause over 33 million acute lower respiratory tract infection episodes in children each year globally.^16^ There is growing evidence, mainly from the economically developed countries, that RSV infection is associated with recurrent wheeze and asthma in the years following the disease.^17^ However, little is known about the impact of RSV infection on child health in economically developing healthcare settings.

Between 2012 and 2015, we conducted the Aetiology of Neonatal Infection in South Asia (ANISA) study in three countries of South Asia (Bangladesh, India, and Pakistan) to measure bacterial and viral infection burden in young infants.^18^ In the ANISA study, we enrolled newborns in their first week of life and followed them up until the age of 59 days. We found RSV as the most frequent cause of neonatal infection (5.4 episodes per 1000 live births).^17^ In this follow-up study (Clinicaltrial.gov, registration ID: NCT03876249), we assessed a nested cohort of children who were part of the ANISA study population to study the association between RSV infection and subsequent chronic respiratory outcomes.

## Methods

We conducted this study in four resource-constrained settings across three countries divided by hostile borders. The Bangladesh site was a rural community located in the northeast of the country in Sylhet district, known as Projahnmo site. In Pakistan, the first site included four urban low-income settlements of Karachi city, and the second site located in Matiari district represented a typical rural agrarian community. The Indian site included two communities in the state of Odisha. The study area in Rourkela was spread over hilly and mining areas with predominantly indigenous tribes. The inhabitants of the Bhubaneswar area were from a rural community.

We hired research assistants (RAs) with a minimum of 12th-grade educational qualifications who visited the households of eligible children. We trained the staff to ensure a uniform implementation process of the study procedures across the study sites. We split the training programmes into two phases. A centralised training was arranged for senior-level project staff, including site-level supervisors, who then cascaded training to the local staff at the respective sites. Participant information sheets (PIS) were translated into the local languages so that participants could easily understand study objectives and procedures.

We enrolled participants at their homes, fearing a low compliance rate if asked to visit the healthcare centres. RAs explained the study procedures to the caregivers (legal guardians) and answered questions before requesting informed consent for their children’s participation in the study. Children whose caregivers agreed to the participation of their children in the study and provided written consent were enrolled in the study. RAs showed the caregivers two videos demonstrating asthma signs and symptoms. One video was developed by the Wellington Asthma Research Group,^19^ and the other video was developed by the RSV consortium in Europe (RESCEU).^20^ RAs also supplied a ‘Child Card’ to the individual caregivers to record the incidence of respiratory-related illness of their children for the next 12 months. Then, they used a structured questionnaire to collect the current and previous respiratory-related disease information, frequency of hospital visits and household demographic information. Asthma history of the enrolled children was collected using the questionnaires designed by the International Study of Asthma and Allergies in Childhood (ISAAC) team.^21^ Over 12 months, RAs visited the children at home on three separate occasions to record respiratory-related illnesses history.

We performed pre- and post-exercise lung function assessments in the children during the clinical assessment visit. We used spirometers (EasyOne^®^, NDD, Zürich, Switzerland) to measure the lung function of the enrolled children, and results were interpreted using the reference value demonstrated by Wang *et al.*^22^ with 10% adjustment. The clinical assessment was conducted in outreach clinics as some of the participants lived in 2- to 3-hour travel distance from the nearest healthcare facilities. In Bangladesh and Pakistan sites, physicians conducted the spirometry, while in India, RAs (with qualification of bachelor’s degree) conducted the test. We arranged a centralised training in Nepal for the lead physicians of each site to demonstrate the spirometry test procedures and study implementation plan. Then, a three-day virtual training was arranged for each site. After the site-specific training, each of the training participants conducted 10 spirometry tests on non-study children aged 7–10 years. Those who performed at least eight acceptable spirometry readouts out of 10 attempts were allowed to assess the study children. A refresher training was arranged for the staff who were unable to conduct eight acceptable spirometry out of 10 attempts. Spirometers that were used in this study had self-quality check feature which graded each attempt as A, B, C, D, or F. Each participant was asked to perform at least three manoeuvres, which the spirometer graded as A, B, or C. If a participant failed to perform three acceptable manoeuvres within a session of a maximum of eight attempts, then the test was rescheduled for another day. Each spirometry readout (spirogram) was shared with the coordination team. A research investigator trained in paediatric spirometry reviewed the individual report to check whether the manoeuvres (1) reached the plateau, (2) were acceptable (no hesitation, no glottis, no coughs during the first second, and continued to the end), and (3) were reproducible (the two largest FVC and FEV1 values were within 10% of the highest value). Spirometry that did not meet these three criteria was repeated.

For the exercise challenge test, we asked the children to run between two bollards placed 10 m apart on a flat surface for 6 minutes. RAs ran with the children to keep them motivated to continue the exercise at their maximum capacity. We used the premises of community clinics and school playgrounds for the tests. Spirometry was performed before and 5 minutes following completion of the exercise. Once the children completed the post-exercise spirometry, a phlebotomist collected a drop of blood from the participants’ fingertips to prepare thin smears that were later read under a light microscope to enumerate eosinophilic granulocytes.

## Results

**Figure 1.**
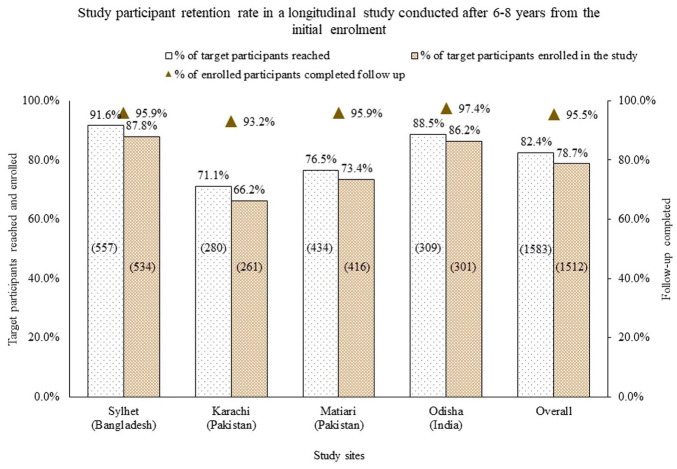
Success in participants tracking and enrolment after 6–8 years from the initial enrolment for a longitudinal cohort study in South Asia.

We planned to follow a total of 1921 children at four study sites. Of these, 75 (3.9%) children had died before the follow-up visits, and 263 (13.7%) children migrated from the study areas. Subsequently, we approached caregivers of 1583 (82.4%) children to participate in this study and 1512 (95.5%) provided consent for their children’s participation in the study. There was site-specific variation in the success of reaching the target participants. At the Bangladesh site, 91.6% (557/608) of the target children could be reached compared to 71.1% (280/394) children at the Karachi site (Figure 1). There was no significant inter-site difference in receiving consent from the caregivers who were approached. Among the enrolled children, 1476 (97.6%) completed the 12-month follow-up visits.

### Clinical assessment of the participants

**Figure 2.**
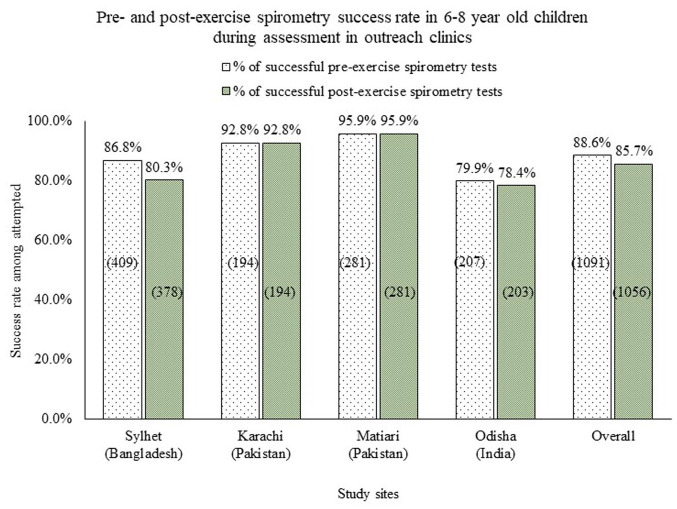
Success in performing pre- and post-exercise spirometry in outreach clinics on 6–8 years old children in three South Asian countries.

A total of 1232 (81.5%) children participated in the clinical assessment process. Among them, 1091 (88.6%) had acceptable pre-exercise spirometry and 1056 (85.7%) had acceptable post-exercise spirometry (Figure 2). A total of 1182 (96.0%) participants provided capillary blood samples for the eosinophil test, and with 1149 (97.2%) of sufficient quality for analysis.

The mean pre-exercise lung forced vital capacity (FVC) of the participants was 1.34 L (SD: 0.35) which was 98% of the mean predicted value. Similarly, the mean forced expiratory volume in the first 1 second (FEV1) and FEV1/FVC ratio were also similar to the mean predicted value (Table 1). At the population level, the mean post-exercise FVC and FEV1 did not differ significantly from their respective pre-exercise values. The post FEV1/FVC ratio was significantly lower (by 0.8%) compared to the pre-FEV1/FVC ratio (Table 1).

**Table 1.** Pre- and post-exercise lung functions of 6–8 years old children.

Parameters	Pre-exercise spirometry (*N* = 1091)		Post-exercise spirometry (*N* = 1057)		Changes	*P* value
	Absolute value, mean (SD)	% Predicted mean, (SD)	Absolute value, mean (SD)	% Predicted, mean (SD)	Mean difference in absolute values, (95% CI)	
FVC (L)	1.34 (0.35)	98.0 (22)	1.33 (0.35)	97.5 (22)	–0.01(–0.04, 0.02)	0.52
FEV1 (L)	1.20 (0.32)	98.6 (22)	1.18 (0.32)	97.4 (23)	–0.02 (–0.05, 0.01)	0.19
FEV1/FVC%	89.6 (6.4)	100 (7.2)	88.8 (6.8)	100 (7.5)	–0.82 (–1.4, –0.26)	<0.01

CI, confidence interval; FEV1, forced expiratory volume in the first 1 second; FVC, forced vital capacity; SD, standard deviation.

### Implementation challenges and adaptation

**Figure 3.**
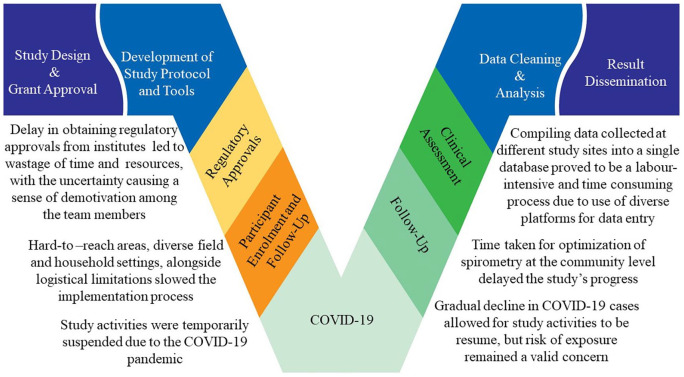
Implementation challenges of asthma assessment in children in South Asia.

Although the research protocol and study tools having been developed in consultation with experts who had years of experience conducting clinical studies in resource-limited settings, we faced multiple challenges during the implementation of the study protocol due to the diverse nature of the field settings and the existing geopolitical conflicts within the three involved countries (Figure 3).

### Protocol and tools development

There was inter-site heterogeneity in respect to ethnicity, religion, languages, and educational qualifications. We formed a coordination team including members from the participating sites and respiratory health experts to guide the protocol adaptation considering the cultural issues and field contexts. We avoided asking any income-related questions or banking information. Instead, we recorded various household elements (for example, primary material of the household walls, roof, and floor where participants lived, and availability of different home appliances) to assess participants’ socio-economic status. We translated the data collection forms into the local languages so that the caregivers could understand the questions correctly and respond accordingly. All forms and questionnaires were piloted at each study site before using these in the study and adapted according to site-specific feedback. This allowed the uniform implementation of study across the study sites.

### Regulatory approval

We required sponsorship approval from the Academic and Clinical Central Office for Research and Development (ACCORD) to start the field operation. The ACCORD sponsorship application package included the ethical approval letters from each of the participating institutes in Bangladesh, India, and Pakistan, who implemented the study in their respective countries. We needed 3–4 months to get the ethical clearance from the partner institutes. Thus, the submission of the sponsorship application to ACCORD was delayed by 6 months. We also required approval from the Indian Health Ministry Screening Committee, which took an additional 8 months. We followed a sequential participant recruitment process to minimise the impact of regulatory delays. Study sites started their field operation after they obtained the required regulatory approvals without waiting for other sites. The first participant at the Bangladesh site was enrolled in September 2019, while the first participant at the India site was enrolled in August 2020. Opening the sites sequentially helped us to maintain the staff motivation and to transfer the knowledge gained from one site to another.

### Participant enrolment and follow-up

It was imperative to have experienced field staff fluent in the local language to conduct household visits as in general respondents had low levels of literacy. We prioritised female staff at the field level and, where possible, from the local residents as female staff had a wider acceptance among the respondents who were mostly the mother of the participants.

As we had not been in touch with the selected children for the last 6–8 years, we initially contacted the families over the phone to collect their updated home addresses and schedule the first visit. Households that could not be reached by telephone were visited up to three times if the caregivers were absent during the previous visits. We asked their neighbours/relatives for updated contact details for the households that had shifted. Through these processes, we could reach 82.4% of the target population. Out-migration (13.7% of the targeted population) and prior deaths (3.9%) were the two primary reasons for non-enrolment. There were site-specific variations in reaching the target participants and enrolment. Bangladesh site had a higher success rate in participant identification and enrolment compared to the other sites. Moving of households resulted in a lower success in reaching the families in Pakistan and India sites.

### Clinical assessment

**Figure 4.**
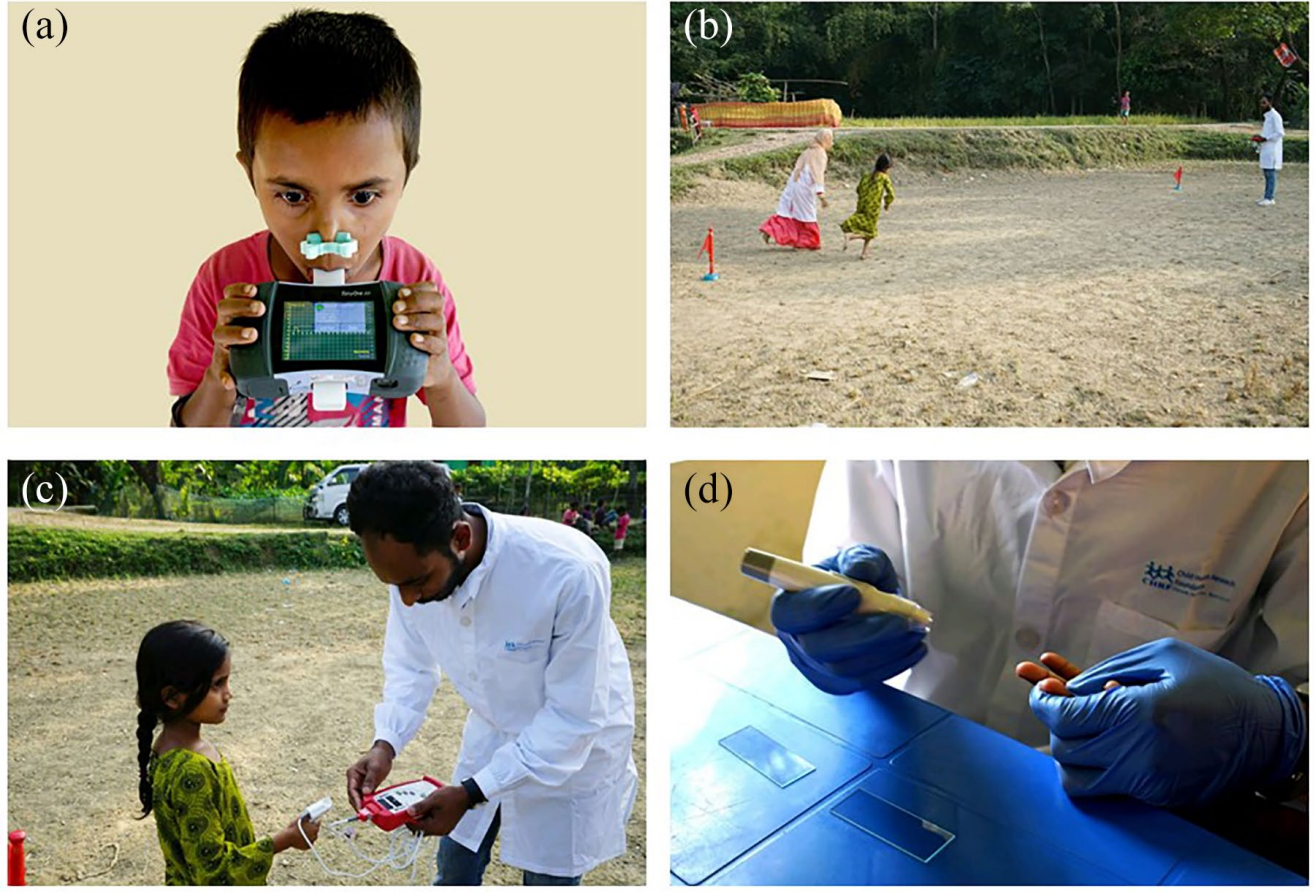
Children assessment at an outreach clinic in a rural community of Bangladesh (a) a participant conducting spirometry test using a portable spirometer, (b) a participant is running in the open air with a research assistant within two bollards placed 10 m apart for exercise challenge test, (c) a team member taking post-exercise heart rate to check whether the exercise was optimal for exercise challenge test, and (d) a phlebotomist taking blood from a participant’s fingertips to count blood eosinophil.

As mentioned earlier, we assessesed the lung functions of the children in outreach clinics to reduce the travel time of the participants (Figure 4). To motivate the children and alleviate their fear and anxiety, we performed the test in the presence of their caregivers and invited two to three children at a time for the assessment and conducted the test one by one in front of the others so that children could learn the techniques by following others.

**Figure 5.**
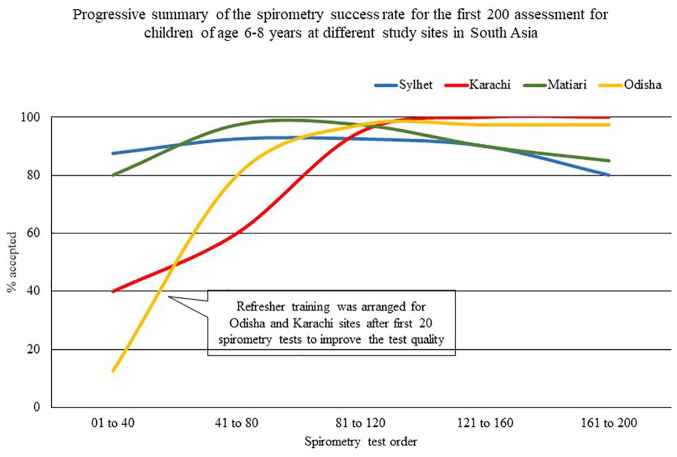
Gradual improvement in performing spirometry tests on 6–8 years old children at different study sites in South Asia.

During the study, it appeared that the spirometry training, which was conducted remotely for Pakistan and Indian sites due to travel restrictions for the COVID-19 pandemic, was suboptimal for the study staff. At these two sites, we had to repeat over 60% of the first 40 spirometry tests as they did not meet the test acceptance criteria. Hence, we arranged an onsite training on spirometry for these two sites with the help of local experts. A physician from the Matiari site spent 5 days with the Karachi team, and in Odisha, we conducted a 3-day training with the support of KEM Hospital Research Centre, Pune. This refresher training helped the sites to improve their spirometry test quality (Figure 5).

In this study, 86% of the children who attended the clinical assessment process completed both pre- and post-exercise spirometry. Performing the procedure at the community level was a challenge as this test was not previously optimised for outreach clinics. We conducted a feasibility test of open-air running exercise on non-study children as we did not have treadmills or cycle ergometers to conduct the exercise challenge test. We asked the participants to complete a 6-minute continuous run with their full capacity and after the run, we measured the heart rate of the participants. A total of 29 children participated in the feasibility test which showed that 86% of the children reached a heart rate of ⩾170 bpm (range: 162–207 bpm) equivalent to  > 85% of maximal heart for their age. Then, we followed this procedure for the exercise challenge test. We found that safe flat surfaces for the exercise were limited at the community level as available fields were mostly uneven and submerged during the rainy season. We used local school playgrounds and courtyards of community health clinics for the exercise but needed to consider the school and clinics’ work hours. Also, a member of the field staff ran with the participant during the test to keep the participants motivated as some participants discontinued the exercise after 1–2 minutes of the run.

In the ANISA study, we had found caregivers as unwilling to provide venous blood from their children. As a result, we planned to count the eosinophil blood cells using peripheral blood films which required only a drop of blood. We collected the blood from the participants’ fingertips using lancets and prepared blood smears over glass slides, which were later examined under light microscopes. We observed a high success rate in collecting blood from the fingertips as 96% of the caregiver agreed to provide blood for the eosinophil test.

### Data management

Data management was a challenge for this multicentre study as sites used different platforms for data entry and storage based on the respective site capacity. Two study sites (Sylhet, Bangladesh and Matiari, Pakistan) collected data on paper-based questionnaires as they operated in remote areas with poor Internet facilities. They used Visual Basic 6.0 for data entry and Microsoft SQL Server 2008 R2 for data storage. The other site in Pakistan (Karachi) used an android-based online platform for data collection, and the Odisha site used REDCap system. While this flexible approach for data entry platforms was helpful for the study sites, the use of multiple platforms resulted in extra workload for the coordination centre to collate the data into a single database for monitoring and analysis. A uniform data entry and storage platform would reduce our workload in collating the site data into a single database.

### Effect of COVID-19 pandemic

The COVID-19 pandemic forced us to suspend study activities multiple times at different study sites which affected the study timelines. We were required to provide refresher training to the field staff before resuming activities and supplied personal protective equipments, including the face masks, gloves, and hand sanitisers, for household visits and clinical assessment. Study staff were required to maintain social distancing and don face masks throughout the data collection process which was uncomfortable for them in a hot humid condition.

## Discussion

**Figure 6.**
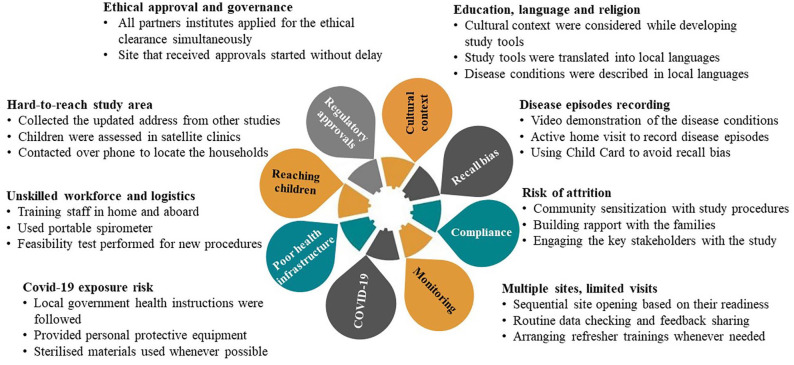
Strategies undertaken to mitigate the implementation challenges in assessment of the long-term impact of RSV infection study in South Asia.

In a longitudinal study, we followed 1512 children for 12 months to record their respiratory health outcomes. We developed a community-based asthma and lung function assessment strategy suitable for LMICs. Participant enrolment and applying the research methodologies was a challenge for us as the children were spread over four vast rural and peri-urban areas in three South Asia counties and had not been contacted for the last 6–8 years. We used multipronged approaches to reach these children and complete the follow-up (Figure 6).

We hired field staff from local residents who could navigate the geographic and cultural contexts and facilitated community understanding of the study procedures and tools. We enrolled the participants at their homes and assessed the children in nearby outreach clinics. Consequently, we could enrol 78.7% of the target population which was comparable with other birth cohort studies conducted in developed countries,^23^ and 81.5% of enrolled children attended the spirometry test which was higher than the studies conducted previously in high-income countries.^24,25^ In this study, 88.6% of the pre-exercise spirometry readouts met the good spirometry test criteria which was consistent with the previous studies conducted on children in developed countries.^26,27^ At the population level, the airway functions (FVC, FEV1, and FEV1/FVC ratio) did not differ from the predicted values which also supports the overall good quality of the spirometry. Forming mobile teams for conducting clinical assessments in the outreach clinics, closely monitoring the data quality, and arranging need-based refresher trainings helped us to conduct spirometry in these resource-constrained areas.

We struggled to initiate the study activities according to the planned schedule due to the delay in attaining regulatory approvals from some authorities, but these were not unexpected as such delays are common for multicentre studies.^28^ While the regulatory processes help improve research quality and protect human rights, delays in receiving regulatory approvals added complexity and uncertainty to our study.^29^ We had to pay staff for several months at the India site without being able to conduct any work due to the delay in attaining the required regulatory approvals to start the field activities. More importantly, such delays led to loss of interest, motivation, and time. This regulatory delay could be shortened if regulatory authorities worked in parallel instead of following a serial process to review the study protocol and ethics documents.

Geopolitical issues still act as a significant barrier to conducting collaborative research in South Asia,^30^ despite the region’s obvious geographic, economic, and cultural inter-dependence. The hostile borders across the participating countries led us to travel to ‘third’ countries for training and meetings, adding time and cost. The fact that our group had previously worked together that helped us to overcome the geopolitical issues, nevertheless, this could be a significant barrier for other studies to deliver planned activities within the scheduled timeframe. Policymakers should improve regional cooperation on mutual interest issues to make the overall research process faster and more productive.

The COVID-19 pandemic forced us to suspend study activities multiple times at different study sites, which affected the study workflow and compliance. A significant number of participants refused the clinical assessment due to fear of COVID-19 transmission which negatively impacted the surveillance outcomes. This study has several other limitations such as 6-month intervals between visits, which could introduce recall bias. We tried to minimise the recall bias by providing a ‘Child Card’ and asked the caregivers to record disease episodes in the card when they occur. However, due to poor literacy, many of the caregivers were unable to record the participants’ disease episodes in the given card. We recorded wheeze episodes based on parental feedback to our queries; however, the reliability of symptom reports is still uncertain. Verifying the wheeze symptoms with trained staff could improve the data quality of our study.

The difficulties we faced in study implementation need to be viewed in the context of this study which was conducted mostly in remote areas; therefore, some of the barriers we faced may not be applicable in well-resourced settings. The cultural context of South Asia may also differ from other LMICs settings, and the process for regulatory approvals may be different for the studies conducted in other parts of the world.

In conclusion, the rate of underdiagnosed asthma in children and adults in LMICs is very high and is often more severe when eventually identified. Thus, better access to diagnostic tools can significantly improve respiratory health in LMICs.^31^ Our study was among a few large community-based studies in developing countries that integrated objective measurements with questionnaire-based responses to assess children’s respiratory health. Scaling up these methods while assessing asthma in children in resource-poor settings can improve accuracy of diagnosis and improve asthma burden estimation at the community level, aiding better decision-making and patient management.

## Declarations

**Ethics approval and consent to participate:** This study was approved by the following ethical review boards: Bangladesh Institute of Child Health (Bangladesh), Aga Khan University (Pakistan), Asia Institute of Public Health (Indian), and KEM Hospital (India). Legal guardians (caregivers) provided written informed consent for their children’s participation in the study.

**Consent for publication:** We can confirm that we have received written consent for publication from the legal guardians (parents) of the two children and the two field workers who participated in the submitted photos (Figure 4(a) and (c)). Consent was voluntary and no payment in the form of cash or gift was made for their participation and consent process. The participants are happy to use their images for knowledge dissemination in the form of textbooks and journal articles.
